# The homeobox gene *CDX2* in colorectal carcinoma: a genetic analysis

**DOI:** 10.1054/bjoc.2000.1544

**Published:** 2001-01

**Authors:** S Sivagnanasundaram, I Islam, I Talbot, F Drummond, J R F Walters, Y H Edwards

**Affiliations:** MRC Human Biochemical Genetics Unit, Biology Department, University College London, Wolfson House, 4 Stephenson Way, London, NW1 2HE; Academic Department of Pathology, St Mark's Hospital, Northwick Park, HA1 3UJ; Gastroenterology Section, Division of Medicine, Imperial College School of Medicine, Hammersmith Hospital, London, W12 0NN

**Keywords:** *caudal* related homeobox gene, *CDX2*, loss of heterozygosity, colorectal carcinoma

## Abstract

Accumulation of mutations in tumour suppressor genes and oncogenes has been proposed to underlie the initiation and progression of sporadic colorectal cancer (CRC). Evidence is accumulating to suggest that the *caudal* homeobox gene *CDX2* is implicated in the pathogenesis of CRC. The CDX2 transcription factor is expressed in intestinal epithelium and is markedly down-regulated in colon tumours. Furthermore, *Cdx2* heterozygous null mice develop multiple intestinal tumours. In this present study, we have investigated *CDX2* as a potential candidate gene for sporadic CRC by a thorough search of all exons and exon/intron boundaries for DNA polymorphisms and rare variants in a panel of CRC tumours. 6 polymorphisms were identified and the haplotypes determined. In addition two rare variants were found, one of which was only identified in DNA from a CRC case. Loss of heterozygosity was observed in 3 out of 28 informative CRC cases. A possible association between particular haplotypes and tumour progression was also suggested by the data. In addition a preliminary analysis of the relative expression of *CDX2* alleles in tumour/normal tissue suggested some variation in the levels, however further analysis is required before any conclusions can be drawn. While *CDX2* mutations predisposing to sporadic CRC have not been identified, this study has established that loss of *CDX2* contributes towards the progression of some sporadic CRC tumours. © 2001 Cancer Research Campaign http://www.bjcancer.com


*British Journal of Cancer* (2001) **84**(2), 218–225
© 2001 Cancer Research Campaign
doi: 10.1054/ bjoc.2000.1544, available online at http://www.idealibrary.com on IDEAL®

http://www.bjcancer.com

### The homeobox gene *CDX2* in colorectal carcinoma: a genetic analysis

**S Sivagnanasundaram[1], I Islam[1], I Talbot[2], F Drummond[1], JRF Walters[3] and YH Edwards[1]**

[1]MRC Human Biochemical Genetics Unit, Biology Department, University College London, Wolfson House, 4 Stephenson Way, London NW1 2HE; [2]Academic Department of Pathology, St Mark's Hospital, Northwick Park, HA1 3UJ; [3]Gastroenterology Section, Division of Medicine, Imperial College School of Medicine, Hammersmith Hospital, London W12 0NN

**Summary** Accumulation of mutations in tumour suppressor genes and oncogenes has been proposed to underlie the initiation and progression of sporadic colorectal cancer (CRC). Evidence is accumulating to suggest that the *caudal* homeobox gene *CDX2* is implicated in the pathogenesis of CRC. The CDX2 transcription factor is expressed in intestinal epithelium and is markedly down-regulated in colon tumours. Furthermore, *Cdx2* heterozygous null mice develop multiple intestinal tumours. In this present study, we have investigated *CDX2* as a potential candidate gene for sporadic CRC by a thorough search of all exons and exon/intron boundaries for DNA polymorphisms and rare variants in a panel of CRC tumours. 6 polymorphisms were identified and the haplotypes determined. In addition two rare variants were found, one of which was only identified in DNA from a CRC case. Loss of heterozygosity was observed in 3 out of 28 informative CRC cases. A possible association between particular haplotypes and tumour progression was also suggested by the data. In addition a preliminary analysis of the relative expression of *CDX2* alleles in tumour/normal tissue suggested some variation in the levels, however further analysis is required before any conclusions can be drawn. While *CDX2* mutations predisposing to sporadic CRC have not been identified, this study has established that loss of *CDX2* contributes towards the progression of some sporadic CRC tumours. © 2001 Cancer Research Campaign http://www.bjcancer.com

**Keywords**: *caudal* related homeobox gene; *CDX2* ; loss of heterozygosity; colorectal carcinoma

Several alternative genetic pathways lead to colorectal cancer (CRC), but in all cases result in the cell escaping from signals which control cell proliferation and differentiation. Approximately 20% of CRC cases arise due to an inherited predisposition. The principal inherited forms are hereditary non-polyposis colorectal cancer (HNPCC) caused by mutations in DNA mismatch repair genes (mainly *hMLH1* and *hMSH2*; Fishel et al, 1993; Leach et al, 1993; Bronner et al, 1994; Papadopoulos et al, 1994; Liu et al, 1996) and familial adenomatous polyposis (FAP), which arises as a result of mutations in the tumour suppressor gene *APC* (Groden et al, 1991; Nishisho et al, 1991). However, the vast majority of CRC cases are sporadic and arise due to random somatic mutations. The genes involved and the order of genetic events in the genesis of sporadic CRC tumours have not yet been elucidated. Several mutations in the *APC* gene have been identified in sporadic colorectal cancer (Cottrell et al, 1992) and somatic mutations in the *hMSH2* and *hMLH1* genes have been identified in a small proportion of sporadic tumours displaying microsatellite instability (Liu et al, 1995; Konishi et al, 1996). Mutations in several other candidate genes, such as the serine/threonine kinase *STK11* (Dong et al, 1998) and the tyrosine kinase *c-SRC* (Irby et al, 1999) are also thought to be predisposing factors in sporadic CRC.

In this paper we have investigated the role of the *caudal* type homeobox gene, *CDX2*, in sporadic colon cancer. CDX2 is a transcription factor known to be crucial to the normal proliferation and differentiation of intestinal epithelial cells (Duluc et al, 1997; Lorentz et al, 1997). In the mouse, *Cdx2* is expressed in the gut from its earliest formation at 8.5 dpc and is confined to the posterior gut endoderm during late development (Beck et al, 1995). In adults *Cdx2* is found exclusively in intestinal epithelial cells, most abundantly in the proximal colon (James and Kazenwadel, 1991; James et al, 1994). Several CDX2 target genes have been identified, such as the small intestine gene sucrase-isomaltase (Suh et al, 1994) and calbindin-D9K (Lambert et al, 1996) and the colonic gene carbonic anhydrase I (Drummond et al, 1996).

*CDX2* has been implicated in disorders involving abnormal intestinal differentiation and neoplasia. Our studies and those of others, have demonstrated that *CDX2* and its target genes are markedly down regulated in many colorectal carcinomas in rodents and humans (Sowden et al, 1993; Ee et al, 1995; Mallo et al, 1997, 1998). In addition, studies of *Cdx2* null mice have shown that while complete deficiency is embryonic lethal, *Cdx2* heterozygous null mice develop multiple intestinal tumours. The polyps are predominantly located in the colon and do not express *Cdx2* from the remaining wild-type allele (Chawengsaksopahak et al, 1997; Beck et al, 1999; Tamai et al, 1999). These observations led to the proposal that the loss of tumour suppressor function of the *CDX2* gene could be involved in tumorigenesis.

The polyps which arise in the *Cdx2* +/– mice are unusual; their gastrointestinal phenotypes appear to vary. Tamai et al (1999) described two forms, cecal/colonic villiform structures and colonic hamartomas similar to the Peutz-Jegher polyps. These structures have been ascribed to a homeotic anterior transformation of the gut mucosa

*Received 26 June 2000*
*Revised 12 September 2000*
*Accepted 14 September 2000*

*Correspondence to:* YH Edwards due to *Cdx2* haplo-insufficiency and bi-allelic *Cdx2* inactivation, respectively. Beck et al (1999) proposed that the colonic lesions were composed of heterotopic stomach and small intestinal mucosa and Chawengsaksopahak et al (1997) described a cell structure very similar to oesophageal epithelium. Homeosis due to *Cdx2* deficiency was proposed as the cause of the abnormal cellular differentiation.

The human *CDX2* gene is localized to chromosome 13q12–13 (German et al, 1994; Drummond et al, 1997) and it is of interest that chromosome 13 abnormalities have been reported in a number of CRC cases (Lothe et al, 1992; Bardi et al, 1995). These factors taken together make *CDX2* a strong candidate for investigation in the context of sporadic CRC. In this paper we report a genetic analysis of *CDX2* in sporadic CRC. All exons and exon/intron boundaries were thoroughly searched for DNA polymorphisms and rare variants in a panel of CRC tumours and normal control samples. Various polymorphisms have been identified which have enabled the assessment of loss of heterozygosity (LOH) in tumours and an evaluation of the relative mRNA expression of both alleles in tumours. In addition associations between genetic variation and tumour progression were looked for.

### MATERIALS AND METHODS

### Subjects

Flash frozen or paraffin-embedded tumour and corresponding normal colon biopsy material from a total of 51 unrelated sporadic CRC cases were collected from St Mark's Hospital and the Hammersmith Hospital. The sporadic CRC cohort comprised 19 females and 32 males ranging in age from 37 to 96 years (mean age = 69 years). 4 of the cases were of Asian origin, while all others were Caucasian. Except in 6 cases, each tumour sample was examined histologically and the degree of dysplasia recorded. 18 were characterized as adenocarcinoma of the rectum, 6 as sigmoid adenocarcinoma and the remainder were grouped together as adenocarcinoma of the colon. 4 tumours showed features similar to gastric mucosa. For 32 tumour/normal pairs the Dukes' stage classification were available, of which 9 were stage A, 12 were stage B, and 11 were stage C. DNA samples from 60 unrelated individuals from the Centre d'Etude du Polymorphisme Humain (CEPH) collection and 50 unrelated British subjects were used as controls.

### DNA and RNA preparation

Normal and tumour biopsy material was ground to a fine powder under liquid nitrogen. The available biopsy material varied considerably in weight but was divided approximately equally and used to prepare DNA and RNA. Genomic DNA was extracted using a procedure based on the protocol described by Goelz et al (1985). Ground tissue was incubated overnight in 500 µl lytic solution (1% SDS, proteinase K (0.5 mg ml-1) in 1 × TE buffer (pH 8.0)) at 52˚C. This was followed by extraction with equal volumes of Tris-saturated phenol (pH 8.0), followed by phenol:chloroform (1:1) and then chloroform. DNA was ethanol precipitated at –20˚C, washed with 70% ethanol, dried and resuspended in 1 × TE buffer. Total RNA was extracted from the ground tissue (between 60 and 100 mg) using 1 ml of RNAzol™ B (Biogenesis Ltd) and homogenizing in a hand-held glass micro-homogenizer, according to the manufacturer's instruction. RNA was precipitated with isopropanol and resuspended in 30 µl of 1 × TE buffer.

### PCR amplification

10 pairs of primers were used to amplify *CDX2* (Table 1). PCRs contained 50 –100 ng of genomic DNA, 0.5 mM dNTPs, 0.5 µM forward and reverse primers and 0.5 units of *Taq* DNA polymerase

**Table 1** Primer pairs used for PCR amplification of the *CDX2* gene

| Primer pair | Region | Primer sequence (5′–3′) | Ta (˚C) | Product size (bp) |
|---|---|---|---|---|
| 1 | 5′-UTR | F: GCCTGTGCGGGTCTTCCCCG<br>R: CACGGAGCTAGGGTACATGC | 60 | 324 |
| 2[c] | Exon 1 | F: GGACTACGGCGGTTACCACG<br>R: CGTAGCCATTCCAGTCCTCC | 59 | 129 |
| 3 | Exon 1 | F: GGAGGACTGGAATGGCTACGC<br>R: TGCTGCGCCGGCTTCCGCATC | 67 | 303 |
| 4 | Exon 1 | F: GCTGCCGCCGAGCAGCTGTC<br>R: CTGTGCGCACTGGACGCGCG | 75 | 178 |
| 5[c] | Exon 2 | F: CAGACTTGGCAGGAACCAGC<br>R: CATGGTCCTCTGCCAGGCAGTG | 65 | 342 |
| 6[c] | Exon 3 | F: TCAGAACCGCAGAGCAAAGGAGAGG<br>R: CTCAGAGGACCTGGCTGAGG | 59[b] | 126 |
| 7 | Exon 3 | F: CTTGAGTCCGGTGTCTTCCCTG<br>R: GTCTGTAGGTCTATGGCTGTG | 56 | 218 |
| 8 | 3′-UTR | F: GCTAGACTCCTCAGGAGAGAC<br>R:AGGCAATGCTTCTGCCAGTCC | 59 | 224 |
| 9 | 3′-UTR | F: ACTGAGGAGACAGAAAGCCTCC[a]<br>R: CCGCAGTGTAAACCTTGCCTG[a] | 55 | 257 |
| 10[c] | 3′-UTR | F: GCTTGAGGCCAAGATGGCTGC<br>R: GCTCTCTAACAAGTCCTGTTC | 51 | 283 |

[a]0.6 µM of primer was used in PCR amplification. [b]PCR was cycled initially at a Ta of 64˚C for 5 cycles and then at 59˚C for 30 cycles. [c]PCR amplification required 5% formamide.

© 2001 Cancer Research Campaign

(Advanced Biotechnology), in either Buffer I or V (Advanced Biotechnology). Primer pairs 2, 5, 6 and 10 required the addition of 5% formamide to the reaction mix to improve specificity. The PCRs were performed at 96°C for 5 min followed by 35 cycles of 96°C for 30 s, Ta°C (see Table 1) for 30 s and 72°C for 30 s. A final step of 72°C for 5 min was also carried out. PCRs using primer pair 6 were cycled initially at a Ta of 64°C for 5 cycles and then at 59°C for 30 cycles.

### cDNA synthesis and PCR amplification

5 µl of total RNA extracted from tumour and normal tissue samples were used to make cDNA. RNA RT-PCR was carried out using random oligonucleotide primers (Amersham Pharmacia Biotech) to generate first strand cDNA. Control RT-PCRs were performed across exons 2 to 4 of the ubiquitously expressed phosphoglucomutase (PGM1) gene to assess the quality and quantity of the cDNA [PGM1 2F: GAAAAATCAAAGCCATTGGTGGG and PGM1 2R: GGCACCGAGTTCTTCACAGAGGAT]. This also served to confirm that there was no contamination of RNA by genomic DNA. The possibility that the RNA samples contained DNA was also checked by RT-PCR in the absence of reverse transcriptase. cDNA PCRs were set up using conditions optimized for the respective primer pair (see above and Table 1). cDNA PCR amplification from exon 1 to exon 2 was performed using the Ex1F: GCTGCCGCCGAGCAGCTGTC and Ex2R: GGATGGTGATGTAGCGACTG primers at an annealing temperature of 61°C.

### Single strand conformation polymorphism (SSCP) analysis

PCR products were prepared for SSCP analysis in formamide dye (98% formamide, 20 mM EDTA, pH 8.3, 0.05% bromophenol blue and 0.05% xylene cyanol) denatured at 95°C for 5 min and rapidly cooled on ice. Samples were analysed by vertical polyacrylamide gel electrophoresis (18 cm × 16 cm × 0.75 mm, Hoefer SE 600 series, Amersham Pharmacia Biotech) in 0.5 × TBE buffer. Each fragment was analysed under a number of electrophoretic conditions: the polyacrylamide gel was either 10% or 12%, with or without glycerol, at either room temperature (RT) or at 4°C. The running conditions were also varied between 100 to 350 V for 2.5 to 17 h. In one case the addition of 20% formamide to the gel (fragment 3) improved band resolution. After electrophoresis, the gels were washed, fixed and stained as described by Harvey et al (1995).

### Sequence analysis

PCR amplified products were sequenced either automatically using the ABI sequencer 377, BigDye or dRhodamine terminators and AmpliTaq DNA polymerase FS (PE Biosystems) or manually using $^{33}$P-labelled ddNTPs and the Thermo Sequenase Radiolabelled Terminator Cycle Sequencing Kit (USB Corporation) to confirm that the correct sequence had been amplified. Products showing variant SSCP banding patterns were also sequenced.

### Phosphoimaging cDNA samples were manually sequenced using $^{33}$P-labelled ddNTPs and the reaction mix resolved by electrophoresis on a 6% denaturing polyacrylamide gel. The gel was dried and exposed to a phospho-imager screen for 1 to 2 h. Signals of individual bands were quantified using the Fuji X-BAS-1000 phosphoimager. The amount of radioactivity, expressed as a photo-stimulated luminescence (PSL) value and density of radioactivity over a fixed area (PSL/mm$^2$) were obtained using the MACBAS software. The relative amounts of the two alleles were determined taking into account the background signal and inter-lane signal variation (see caption to Fig 3).

### Statistical analysis

The significance of differences in allele frequencies between control and CRC patients was assessed using the 2 by m $\chi^2$ test. The Monte Carlo $\chi^2$ 'Clump' test was used to examine the differences in haplotype frequencies between control and patient subjects and to assess the association between haplotype and Dukes' stage assignment (Sham and Curtis, 1995). A three by three $\chi^2$ test was used to test for association between haplotype and Dukes' stage assignment.

### RESULTS

### Sequence of the human *CDX2* gene at exon/intron boundaries

Since genomic sequence for human *CDX2* was not available in current databases or literature we began by determining genomic sequence at each exon/intron boundary. Comparison of the human *CDX2* cDNA sequence (accession number Y13709) with mouse *Cdx2* genomic sequence (accession number U00454) identified the positions of the introns and defined the sizes of the three exons as 541 bp, 146 bp and 252 bp. The sizes are in agreement with those reported by Yagi et al (1999). Primers designed from cDNA sequence close to the predicted splice sites were used to determine intronic sequence adjacent to each exon. The genomic clone, *λgCDX2.3*, which we have described previously (Drummond et al, 1997) was used as template for this analysis. The exon/intron boundary sequence information compiled in our study has been deposited in the EMBL database (accession numbers Y13709, AJ278431, AJ278432 and AJ278434) and used to design 10 pairs of primers across the *CDX2* gene which amplify the 5′ and 3′-UTR regions, coding sequences and exon/intron splice sites (Table 1).

### Polymorphisms in *CDX2*

PCR amplified products (Table 1) from DNA samples were screened for polymorphisms using a combination of non-radioactive SSCP analysis and direct sequencing.

6 polymorphisms and one rare variant (C1769T in the 3′-UTR) were identified; all are due to point mutations (Figure 1). Two adjacent bases in the 3′ end of intron 1, -32 and -31, were both polymorphic, C-32T and A-31T (primer pair 5). These variants gave rise to six SSCP patterns (Figure 1). Sequence analysis showed that 3 of these could be ascribed to homozygosity at both sites, -32C/-31T; -32T/-31T; -32C/-31A, while the others were due to heterozygous combinations -32CT/-31AT; -32C/-31AT; -32CT/-31T. Two other variant sites T1239C and G1314T were also found together in a single PCR product encompassing part of exon 3 (primer pair 7) and these in combination gave rise to 3 SSCP banding patterns (Figure 1). Sequence analysis showed that two of these patterns could be explained by homozygosity at both sites 1239T/1314G and

 © 2001 Cancer Research Campaign

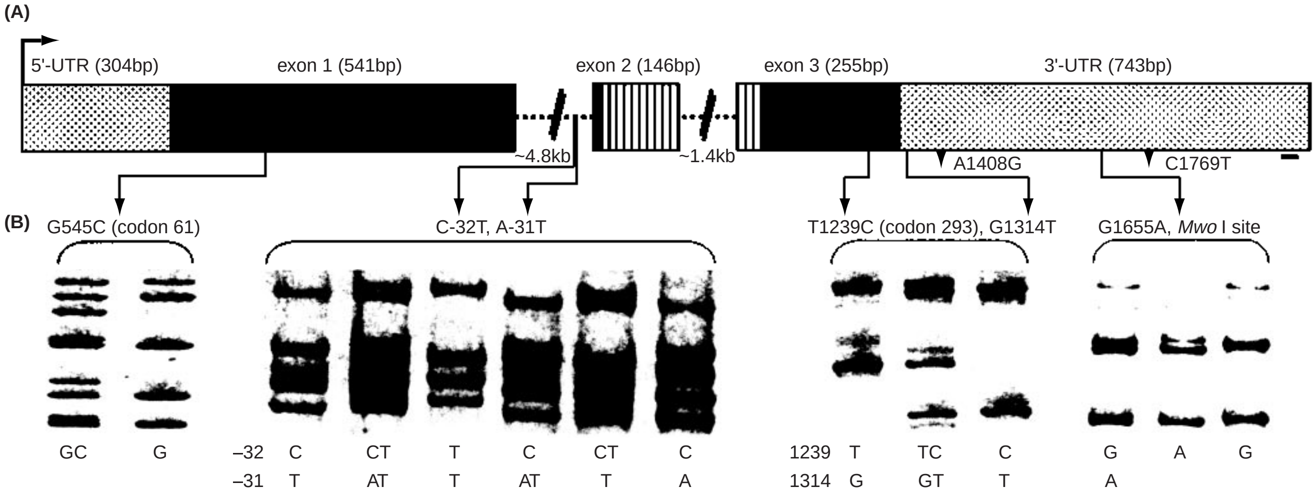

**Figure 1** Structure of the human *CDX2* gene. (**A**) A schematic representation of the genomic structure of the human *CDX2* gene. Solid box – coding exons; speckled box – non-coding regions; hatched box – homeobox (183bp); dotted line – introns, approximate sizes of introns in kb as estimated by Yagi et al, 1999; the transcription start site is indicated by the arrow in the 5′-UTR; the location of the polyadenylation signal is indicated by a bar beneath the 3′-UTR.
(**B**) Polymorphisms and rare variants identified in the human *CDX2* gene in the course of this study. For each polymorphism (indicated by arrows) SSCP banding patterns are depicted and the genotype shown. The location of two rare variants are indicated by arrowheads (SSCP not shown)

1239C/1314T. The third pattern was due to a heterozygous combination of alleles 1239TC/1314GT. The T1239C change leads to a Ser to Pro substitution at codon 293. This codon is Pro in both mice and hamster (German et al, 1992; James et al, 1994; Suh et al, 1994). A further variant G1655A (primer pair 9) was found in the 3′-UTR which leads to a loss of an *Mwo* I site. Finally a silent base change G545C (primer pair 2) was found in exon 1 at codon 61.

Allele frequencies were estimated for all 6 variants in cohorts of control and patient DNA derived from normal tissue biopsy (Table 2). $\chi^2$ tests found no statistically significant difference between control and patient allele frequencies ($P > 0.05$).

The polymorphisms C-32T and A-31T lie in a potential RNA splicing branch site (TTGCAGT) upstream of the splice acceptor site of exon 2. Since mutations in branch sites are known to alter RNA splicing (e.g. BRCA1; Li et al, 1999) we investigated whether these mutations affected splicing at the exon1/exon2 boundary. PCRs across exon1 to exon2 were carried out using cDNA prepared from normal tissues representing the 6 different genotypes (3 of each, see above). A single amplified product of expected size (190 bp) was obtained in all cases and sequencing of this product confirmed that there were no changes in mRNA splicing.

Within the *CDX2* coding exons there are 3 regions of imperfect trinucleotide repeats encoding a stretch of 8 polyalanine, 13 polyhistidine/proline and 7 polyglutamine residues. Though imperfect these regions were searched for variations in repeat numbers but none was found.

The G545C and T1239C polymorphisms have recently been described by Yagi et al (1999) and Wicking et al (1998). The frequency of the 545C variant in the Japanese population (Yagi et al, 1999) was higher (0.11 sporadic CRC samples; 0.07 controls) than in our UK population (0.04 CRC cases; 0.06 controls) while the frequency of the 1239C variant was very similar in both populations. It was not possible to estimate frequencies for the 545C and 1239C in the Australian population from the data reported by Wicking et al (1998). The Lys164 and Leu260 variants which have been reported in the Japanese population with a frequency of around 0.01, were not found in the UK population. As in our study of UK sporadic CRC cases, the Yagi et al (1999) study also found no difference between control and patient allele frequencies.

### Mutations in *CDX2*

In addition to screening for polymorphic variations, the DNA from the CRC tumours ($n = 51$) was thoroughly analysed for rare mutations by SSCP. Only one rare variant, an A1408G substitution in the 3′-UTR (primer pair 8), was found in a tumour sample that showed features of gastric mucosa. This variation was also present in the DNA from the normal adjacent tissue but was not seen in 47 controls.

### Haplotype analysis and loss of heterozygosity

Amongst the 51 patients with CRC, 42 were genotyped for all 6 polymorphic sites. 13 were homozygous at all 6 sites and could be

**Table 2** Variant allele frequencies at the six polymorphic sites in control subjects and sporadic CRC cases

| Polymorphisms | Alleles | Controls | | CRC cases |
|---|---|---|---|---|
| | | CEPH ($n$ = 60) | British ($n$ = 50) | normal tissue ($n$ = 51) |
| G545C, codon 61 | C | 0.04 | 0.06 | 0.04 |
| C-32T | T | 0.33 | 0.27 | 0.38 |
| A-31T | T | 0.78 | 0.83 | 0.80 |
| T1239C, codon 293 | C | 0.16 | 0.10 | 0.18 |
| G1314T | T | 0.16 | 0.10 | 0.18 |
| G1655A, *Mwo* I site | A | 0.47 | 0.49 | 0.40 |

**Table 3** Frequency of haplotypes in CRC and control subjects

| | Haplotypes | | | | | | CRC chromosomes | Normal chromosomes | |
|---|---|---|---|---|---|---|---|---|---|
| | P1 | P2 | P3 | P4 | P5 | P6 | | CEPH | British controls |
| I | G | T | T | T | G | G | 31 (37) | 25 (26) | 18 (29) |
| II | G | C | T | T | G | A | 28 (33) | 37 (39) | 32 (52) |
| III | G | C | A | C | T | G | 14 (17) | 16 (17) | 08 (13) |
| IV | G | C | A | T | G | G | 02 (2) | 08 (8) | – |
| V | G | T | T | T | G | A | 01 (1) | 02 (2) | – |
| VI | G | C | T | T | G | G | 03 (4) | – | 01 (2) |
| VII | G | C | A | C | T | A | – | 01 (1) | – |
| VIII | C | C | T | T | G | A | 03 (4) | 05 (5) | 03 (5) |
| IX | G | C | A | T | G | A | 02 (2) | 02 (2) | – |
| | Total | | | | | | 84 | 96 | 62 |

P1 to P6 represents the following polymorphic sites: G545C; C-32T; A-31T; T1239C; G1314T; G1655A. The haplotypes have been assigned Roman numerals I to IX. Percentage frequency is given in brackets.

categorized into 3 common haplotypes (haplotype I, II and III; Table 3). Genotype analysis of the heterozygous cases revealed that they could be explained either as a combination of common haplotypes or as a combination of one of the common haplotypes with a rare haplotype. In this way complete haplotypes were determined for 84 CRC chromosomes and 158 chromosomes from CEPH and British controls. 6 rare haplotypes were identified in total (IV–IX). Haplotypes I and II accounted for approximately 70% of all chromosomes in the control and CRC patient groups. The observed differences in haplotype frequencies were not statistically significant ($P > 0.05$) when results of a 2 by m table, a collapsed table and a clumped 2 by 2 table were analysed.

28 of the tumour/normal pairs were informative for LOH, that is the normal sample was heterozygous for at least one polymorphic site. Complete LOH was detected in 3 tumour samples: in one case there was loss of haplotype II from a I/II heterozygote, in another, loss of VI from a II/VI heterozygote while the other was loss of I, from a I/II heterozygote (Figure 2A, B, C). The II/VI LOH sample was from a tumour with gastric mucosa phenotype. It is assumed that these allelic losses represent a loss of a moderate to large chromosomal fragment, but since none of these individual LOH cases were heterozygous at both of the two outer polymorphic sites, G545C and G1655A, it is impossible to determine whether there is loss of the entire *CDX2* gene.

### Clinicopathological correlation with haplotype

Association between haplotype and the Dukes' stage assignment of the tumours was investigated (Table 4). A three by three $\chi^2$ test was performed by pooling data for haplotypes III to IX eliminating the large number of cells with expected frequencies of less than 5 (16% of cells with $E < 5$). A significant association was observed ($P = 0.0066$, $\chi^2 = 14.22$, df = 4). This can be attributed to the high frequency of haplotype I in Dukes' stage C cases, haplotype II in Dukes' stage A cases and the remaining rare haplotypes, when pooled together, in Dukes' stage B cases (see Table 4). It is difficult to be certain how much value to put upon this finding since the numbers involved are small and the haplotypes do not vary in their amino acid coding potential. However, further analysis using the Monte Carlo 'Clump' test but excluding the intermediate class, stage B samples (where there might be effects due to grouping of rare haplotypes) generated a $P$ value of 0.027 for the collapsed data. It is relevant to note that while the difference in distribution of haplotypes between CRC cases and controls is not statistically significant there is a greater frequency of haplotype I amongst CRC cases (37%) than controls (CEPH 26%, British controls 29%; Table 3).

No specific association was observed between haplotypes or genotypes and the 4 cases with a tumour of gastric mucosa phenotype, which were haplotypes I/II, II/VI, II/II and III/IV.

**Table 4** Haplotype frequency in CRC individuals assigned with Dukes' stage classification

| | Haplotype | | | | | | Dukes' stage classification | | |
|---|---|---|---|---|---|---|---|---|---|
| | P1 | P2 | P3 | P4 | P5 | P6 | A | B | C (C, C1 and C2) |
| I | G | T | T | T | G | G | 03 (21.4) | 05 (27.8) | 13 (59.1) |
| II | G | C | T | T | G | A | 09 (64.3) | 05 (27.8) | 06 (27.3) |
| III | G | C | A | C | T | G | 01 (7.15) | 03 (16.7) | 02 (9.1) |
| IV | G | C | A | T | G | G | – | 01 (5.6) | – |
| V | G | T | T | T | G | A | – | – | 01 (4.5) |
| VI | G | C | T | T | G | G | 01 (7.15) | – | – |
| VIII | C | C | T | T | G | A | – | 02 (11.1) | – |
| IX | G | C | A | T | G | A | – | 02 (11.1) | – |
| | Total | | | | | | 14 | 18 | 22 |

P1 to P6 represents the following polymorphic sites: G545C; C-32T; A-31T; T1239C; G1314T; G1655A. The haplotypes have been assigned Roman numerals I to IX. Percentage frequency is given in brackets.

 *© 2001 Cancer Research Campaign*

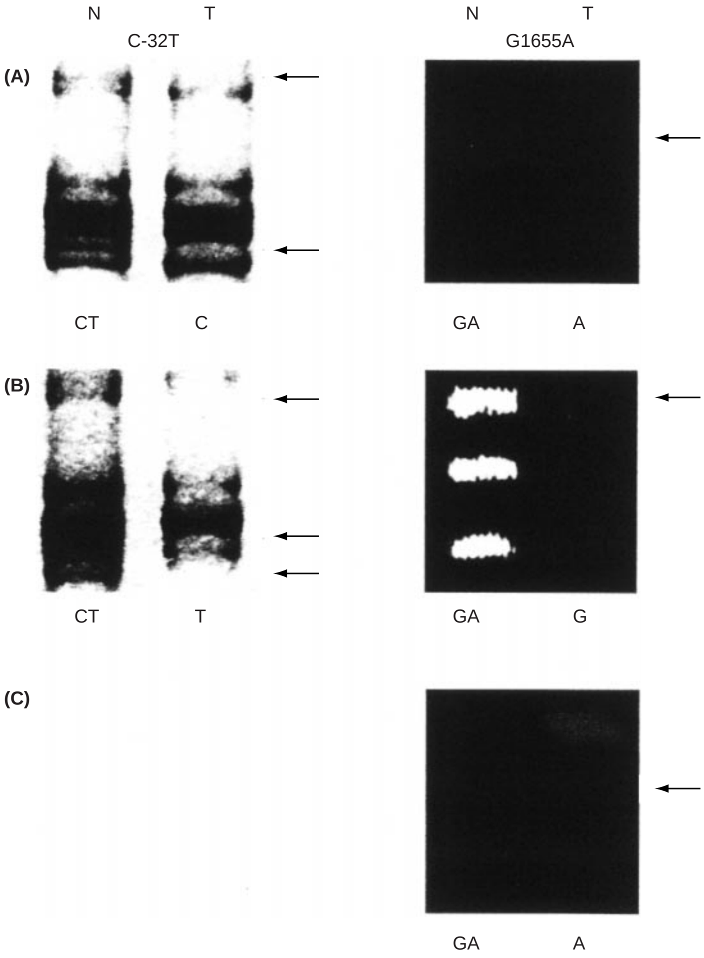

**Figure 2** LOH analysis. Results depicting the LOH observed at polymorphic sites C-32T (shown as SSCP banding patterns) and G1655A (shown as *Mwo* I digest) in three CRC tumour (T) tissue (**A**, **B**, **C**). The genotypes of the tumour and normal (N) DNA are shown below the corresponding SSCP and *Mwo* I digest. The position of the bands corresponding to lost alleles are indicated with arrows

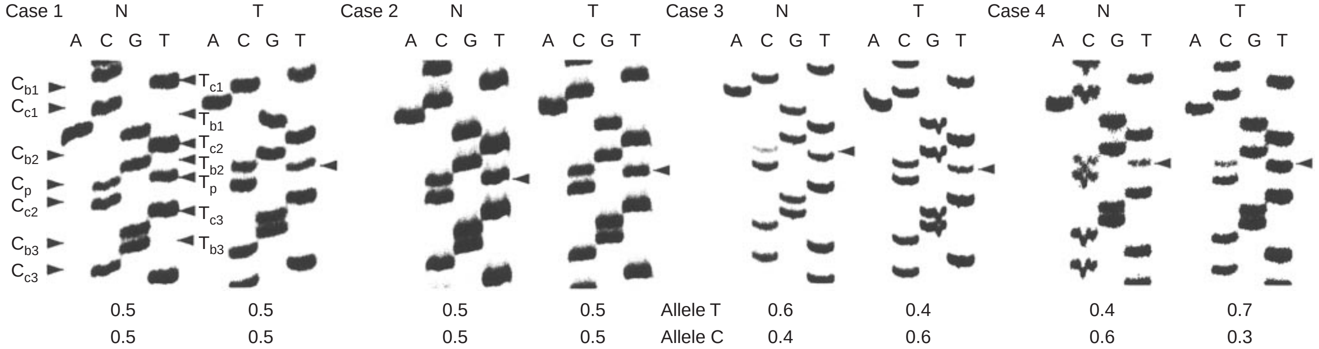

**Figure 3** Relative expression of normal and variant alleles at the T1239C polymorphic site in 4 normal (N)/tumour (T) tissue pairs. The relative proportion contributed by each allele, was determined using the following formula: $\%C = x(C_p - C_b)/[(T_p - T_b) + x(C_p - C_b)]$, where $T_p$ and $C_p$ are the PSL/mm$^2$ values of the normal and variant allele bands. $T_b$ and $C_b$ are the average of three background PSL/mm$^2$ readings (e.g. $C_{b1}$, $C_{b2}$, $C_{b3}$) and $T_c$ and $C_c$ are the average of three non-variant C or T PSL/mm$^2$ readings (e.g. $C_{c1}$, $C_{c2}$, $C_{c3}$) from the appropriate lane. x is the control factor for gel lane to lane variation and is determined using the following equation: $(T_c - T_b)/(C_c - C_b)$

### Relative expression of common and variant alleles

In addition to searching for mutations, we have explored the possibility of assessing the relative expression of pairs of alleles in mRNA derived from biopsy samples from individuals heterozygous for *CDX2* polymorphisms. Differences in the relative allelic expression in the tumour sample might indicate the presence of mutations affecting transcription. The T1239C polymorphism was used as a marker in this preliminary investigation of 7 CRC cases from whom biopsy mRNA was prepared. Common and variant alleles at this site were assessed semi-quantitatively by sequence analysis and phosphoimaging using a procedure (formulated in the caption to Figure 3) which takes into account the background PSL/mm$^2$ reading at 3 blank positions and at 3 non-variant, control, bands in each lane. In this way appropriate correction for background signal intensity and gel lane to lane variation was made. In addition the relative levels in the tumour versus the normal mRNA samples were compared. Some variation in the estimates of expression was seen in all samples tested but all fell within the range of 0.3 to 0.7 (expected is 0.5 where both alleles are equally expressed). Typical analyses for 4 tumour/normal pairs are shown in Figure 3. In case 4, one of the pair of alleles is at a relatively low level (allele C = 0.3) in the tumour sample only, and it is tempting to speculate that this might indicate the presence of unidentified sequence variation affecting gene transcription. It would be unwise to draw conclusions from this limited data set, however, our data suggests that this is a technique worth exploring further in the context of cancer aetiology.

### DISCUSSION

In this study, we have investigated the *caudal*-type homeobox gene, *CDX2*, as a potential candidate gene in sporadic colorectal tumorigenesis. We identified 6 sequence polymorphisms in the human *CDX2* gene; however there were no differences in the frequency of variant alleles between controls and CRC cases. Evidence has emerged which suggests that *CDX2* plays a contributory role in tumour progression although there is no evidence for a significant role in tumour initiation. LOH was found in 3 out of 28 informative cases in our study of UK CRC cases. This incidence of LOH is similar to that described for Japanese sporadic CRC cases (2/20, Yagi et al, 1999). If our data and those of Yagi et al (1999) are combined it appears that approximately 10% of CRC tumour progression involves the loss of *CDX2*.

In addition, a statistically significant association was found between Dukes' stage histopathology and *CDX2* haplotype which suggests that haplotype I could be a predisposing factor for advanced tumour progression. It is clearly important to verify this finding in a larger tumour cohort. It is unfortunate that although the Dukes' stages were recorded for the tumour cohort examined by Wicking et al (1998), *CDX2* haplotypes were not derived and association with tumour progression was not investigated. The association between haplotype and tumour progression, if real, must be due to mutations which affect the level of cellular CDX2, although the difference in levels might be only marginal. There is evidence from studies of mice carrying the *Cdx2* null alleles of a dose-dependent response to Cdx2 transcriptional regulatory activity at the cellular level. It can be envisaged that mutations that subtly alter the activity of Cdx2 may not be significant in the normal epithelial cell but fast dividing neoplastic cells may be much more vulnerable to small changes in Cdx2 levels. One interpretation of our association study is that the presence of haplotype II may predict a less aggressive tumour and a better outcome for the patient while the presence of haplotype I predicts a more invasive tumour and worse outcome. If these mutations are features of particular haplotypes then they must lie in sequences outside those examined here, since the sequence variants which comprise the haplotypes appear not to affect RNA structure or protein function.

It was of particular interest to investigate those tumours in our patient set that showed a gastric mucosa phenotype since their appearance is similar to that of the heterotopic polyps found in the *Cdx2* heterozygous knockout mice. One of the 4 showed LOH of *CDX2* and another a rare variant in the 3′-UTR of *CDX2*. While the significance of these observations is uncertain they are nevertheless intriguing.

### ACKNOWLEDGEMENT

SS was supported by a project grant (Reference: SP2459/0101) from the Cancer Research Campaign.

### REFERENCES

Bardi G, Sukhikh T, Pandis N, Fenger C, Kronborg O and Heim S (1995) Karyotypic characterization of colorectal adenocarcinomas. *Genes Chromosomes Cancer* **12(2)**: 97–109

Beck F, Erler T, Russell A and James R (1995) Expression of Cdx-2 in the mouse embryo and placenta: possible role in patterning of the extra-embryonic membranes. *Dev Dyn* **204(3)**: 219–227

Beck F, Chawengsaksophak K, Waring P, Playford RJ and Furness JB (1999) Reprogramming of intestinal differentiation and intercalary regeneration in *CDX2* mutant mice. *Proc Natl Acad Sci* **96(13)**: 7318–7323

Bronner CE, Baker SM, Morrison PT, Warren G, Smith LG, Lescoe MK, Kane M, Earabino C, Lipford J, Lindblom A, et al (1994) Mutation in the DNA mismatch repair gene homologue hMLH1 is associated with hereditary non-polyposis colon cancer. *Nature* **368**: 258–261

Chawengsaksophak K, James R, Hammond VE, Kontgen F and Beck F (1997) Homeosis and intestinal tumours in *CDX2* mutant mice. *Nature* **386**: 84–87

Cottrell S, Bicknell D, Kaklamanis L and Bodmer WF (1992) Molecular analysis of APC mutations in familial adenomatous polyposis and sporadic colon carcinomas. *Lancet* **340**: 626–630

Dong SM, Kim KM, Kim SY, Shin MS, Na EY, Lee SH, Park WS, Yoo NJ, Jang JJ, Yoon CY, Kim JW, Kim SY, Yang YM, Kim SH, Kim CS and Lee JY (1998) Frequent somatic mutations in serine/threonine kinase 11/Peutz-Jeghers syndrome gene in left-sided colon cancer. *Cancer Res* **58(17)**: 3787–3790

Drummond F, Sowden J, Morrison K and Edwards YH (1996) The caudal-type homeobox protein Cdx-2 binds to the colon promoter of the carbonic anhydrase 1 gene. *Eur J Biochem* **236(2)**: 670–681

Drummond F, Putt W, Fox M and Edwards YH (1997) Cloning and chromosome assignment of the human *CDX2* gene. *Ann Hum Genet* **61**: 393–400

Duluc I, Lorentz O, Fritsch C, Leberquier C, Kedinger M and Freund JN (1997) Changing intestinal connective tissue interactions alters homeobox gene expression in epithelial cells. *J Cell Sci* **110**: 1317–1324

Ee HC, Erler T, Bhathal PS, Young GP and James R (1995) *CDX2* homeodomain expression in human and rat colorectal adenoma and carcinoma. *Am J Pathol* **147**: 586–592

Fishel R, Lescoe MK, Rao MR, Copeland NG, Jenkins NA, Garber J, Kane M and Kolodner R (1993) The human mutator gene homolog MSH2 and its association with hereditary nonpolyposis colon cancer [published erratum appears in Cell 1994 Apr 8;77(1):167]. *Cell* **75(5)**: 1027–1038

German MS, Wang J, Chadwick RB and Rutter WJ (1992) Synergistic activation of the insulin gene by a LIM-homeo domain protein and a basic helix-loop-helix protein: building a functional insulin minienhancer complex. *Genes Dev* **6(11)**: 2165–2176

German MS, Wang J, Fernald AA, Espinosa R, Le Beau MM and Bell GI (1994) Localization of the genes encoding two transcription factors, LMX1 and CDX3, regulating insulin gene expression to human chromosomes 1 and 13. *Genomics* **24(2)**: 403–404

Goelz SE, Hamilton SR and Vogelstein B (1985) Purification of DNA from formaldehyde fixed and paraffin embedded human tissue. *Biochem Biophys Res Commun* **130(1)**: 118–126

Groden J, Thliveris A, Samowitz W, Carlson M, Gelbert L, Albertsen H, Joslyn G, Stevens J, Spirio L, Robertson M, et al (1991) Identification and characterisation of the familial adenomatous polyposis coli gene. *Cell* **66(3)**: 589–600

Harvey CB, Pratt WS, Islam I, Whitehouse DB and Swallow DM (1995) DNA polymorphisms in the lactase gene. Linkage disequilibrium across the 70-kb region. *Eur J Hum Genet* **3(1)**: 27–41

© 2001 Cancer Research Campaign

Irby RB, Mao W, Coppola D, Kang J, Loubeau JM, Trudeau W, Karl R, Fujita DJ, Jove R and Yeatman TJ (1999) Activating SRC mutation in a subset of advanced human colon cancers. *Nat Genet* **21**: 187–190

James R and Kazenwadel J (1991) Homeobox gene expression in the intestinal epithelium of adult mice. *J Biol Chem* **266(5)**: 3246–3251

James R, Erler T and Kazenwadel J (1994) Structure of the murine homeobox gene *CDX2*. Expression in embryonic and adult intestinal epithelium. *J Biol Chem* **269**: 15229–15237

Konishi M, Kikuchi-Yanoshita R, Tanaka K, Muraoka M, Onda A, Okumura Y, Kishi N, Iwama T, Mori T, Koike M, Ushio K, Chiba M, Nomizu S, Konishi F, Utsunomiya J and Miyaki M (1996) Molecular nature of colon tumors in hereditary nonpolyposis colon cancer, familial polyposis, and sporadic colon cancer. *Gastroenterology* **111(2)**: 307–317

Lambert M, Colnot S, Suh E, L'Horset F, Blin C, Calliot ME, Raymondjean M, Thomasset M, Traber PG and Perret C (1996) cis-Acting elements and transcription factors involved in the intestine-specific expression of the rat calbindin-D9K gene: binding of the intestine-specific transcription factor Cdx-2 to the TATA box. *Eur J Biochem* **236(3)**: 778–788

Leach FS, Nicolaides NC, Papadopoulos N, Liu B, Jen J, Parsons R, Peltomaki P, Sistonen P, Aaltonen LA, Nystrom-Lahti M, et al (1993) Mutations of a mutS homolog in hereditary nonpolyposis colorectal cancer. *Cell* **75(6)**: 1215–1225

Li SS, Tseng HM, Yang TP, Liu CH, Teng SJ, Huang HW, Chen LM, Kao HW, Chen JH, Tseng JN, Chen A, Hou MF, Huang TJ, Chang HT, Mok KT and Tsai JH (1999) Molecular characterization of germline mutations in the BRCA1 and BRCA2 genes from breast cancer families in Taiwan. *Hum Genet* **104(3)**: 201–204

Liu B, Nicolaides NC, Markowitz S, Willson JK, Parsons RE, Jen J, Papadopolous N, Peltomaki P, de-la-Chapelle A, Hamilton SR et al (1995) Mismatch repair gene defects in sporadic colorectal cancers with microsatellite instability. *Nat Genet* **9(1)**: 48–55

Liu B, Parsons R, Papadopoulos N, Nicolaides NC, Lynch HT, Watson P, Jass JR, Dunlop M, Wyllie A, Peltomaki P, de-la-Chapelle A, Hamilton SR, Vogelstein B and Kinzler KW (1996) Analysis of mismatch repair genes in hereditary non-polyposis colorectal cancer patients. *Nat Med* **2(2)**: 169–174

Lorentz O, Duluc I, Arcangelis AD, Simon-Assmann P, Kedinger M and Freund JN (1997) Key role of the *CDX2* homeobox gene in extracellular matrix-mediated intestinal cell differentiation. *J Cell Biol* **139(6)**: 1553–1565

Lothe RA, Fossli T, Danielsen HE, Stenwig AE, Nesland JM, Gallie B and Borresen AL (1992) Molecular genetic studies of tumor suppressor gene regions on chromosomes 13 and 17 in colorectal tumors. *J Natl Cancer Inst* **84(14)**: 1100–1108

Mallo GV, Rechreche H, Frigerio JM, Rocha D, Zweibaum A, Lacasa M, Jordan BR, Dusetti NJ, Dagorn JC and Iovanna JL (1997) Molecular cloning, sequencing and expression of the mRNA encoding human Cdx1 and Cdx2 homeobox. Down-regulation of Cdx1 and Cdx2 mRNA expression during colorectal carcinogenesis. *Int J Cancer* **74(1)**: 35–44

Mallo GV, Soubeyran P, Lissitzky JC, Andre F, Farnarier C, Marvaldi J, Dagorn JC and Iovanna JL (1998) Expression of the Cdx1 and Cdx2 homeotic genes leads to reduced malignancy in colon cancer-derived cells. *J Biol Chem* **273(22)**: 14030–14036

Nishisho I, Nakamura Y, Miyoshi Y, Miki Y, Ando H, Horii A, Koyama K, Utsunomiya J, Baba S and Hedge P (1991) Mutations of chromosome 5q21 genes in FAP and colorectal cancer patients. *Science* **253**: 665–669

Papadopoulos N, Nicolaides NC, Wei YF, Ruben SM, Carter KC, Rosen CA, Haseltine WA, Fleischmann RD, Fraser CM, Adams MD et al (1994) Mutation of a mutL homolog in hereditary colon cancer. *Science* **263**: 1625–1629

Sham PC and Curtis D (1995) Monte Carlo tests for associations between disease and alleles at highly polymorphic loci. *Ann Hum Genet* **59**: 97–105

Sowden J, Leigh S, Talbot I, Delhanty J and Edwards Y (1993) Expression from the proximal promoter of the carbonic anhydrase 1 gene as a marker for differentiation in colon epithelia. *Differentiation* **53(2)**: 67–74

Suh E, Chen L, Taylor J and Traber PG (1994) A homeodomain protein related to caudal regulates intestine-specific gene transcription. *Mol Cell Biol* **14**: 7340–7351

Tamai Y, Nakajima R, Ishikawa T, Takaku K, Seldin MF and Taketo MM (1999) Colonic hamartoma development by anomalous duplication in *CDX2* knockout mice. *Cancer Res* **59(12)**: 2965–2970

Wicking C, Simms LA, Evans T, Walsh M, Chawengsaksophak K, Beck F, Chenevix-Trench G, Young J, Jass J, Leggett B and Wainwright B (1998) *CDX2*, a human homologue of Drosophila caudal, is mutated in both alleles in a replication error positive colorectal cancer. *Oncogene* **17(5)**: 657–659

Yagi OK, Akiyama Y and Yuasa Y (1999) Genomic structure and alterations of homeobox gene *CDX2* in colorectal carcinomas. *Br J Cancer* **79(3–4)**: 440–444

*© 2001 Cancer Research Campaign* 

## References

[PDF_00617] Bardi G., Sukhikh T., Pandis N., Fenger C., Kronborg O., Heim S. (1995). Karyotypic characterization of colorectal adenocarcinomas.. Genes Chromosomes Cancer.

[PDF_00623] Beck F., Chawengsaksophak K., Waring P., Playford R. J., Furness J. B. (1999). Reprogramming of intestinal differentiation and intercalary regeneration in Cdx2 mutant mice.. Proc Natl Acad Sci U S A.

[PDF_00620] Beck F., Erler T., Russell A., James R. (1995). Expression of Cdx-2 in the mouse embryo and placenta: possible role in patterning of the extra-embryonic membranes.. Dev Dyn.

[PDF_00626] Bronner C. E., Baker S. M., Morrison P. T., Warren G., Smith L. G., Lescoe M. K., Kane M., Earabino C., Lipford J., Lindblom A. (1994). Mutation in the DNA mismatch repair gene homologue hMLH1 is associated with hereditary non-polyposis colon cancer.. Nature.

[PDF_00630] Chawengsaksophak K., James R., Hammond V. E., Köntgen F., Beck F. (1997). Homeosis and intestinal tumours in Cdx2 mutant mice.. Nature.

[PDF_00632] Cottrell S., Bicknell D., Kaklamanis L., Bodmer W. F. (1992). Molecular analysis of APC mutations in familial adenomatous polyposis and sporadic colon carcinomas.. Lancet.

[PDF_00635] Dong S. M., Kim K. M., Kim S. Y., Shin M. S., Na E. Y., Lee S. H., Park W. S., Yoo N. J., Jang J. J., Yoon C. Y. (1998). Frequent somatic mutations in serine/threonine kinase 11/Peutz-Jeghers syndrome gene in left-sided colon cancer.. Cancer Res.

[PDF_00642] Drummond F., Putt W., Fox M., Edwards Y. H. (1997). Cloning and chromosome assignment of the human CDX2 gene.. Ann Hum Genet.

[PDF_00639] Drummond F., Sowden J., Morrison K., Edwards Y. H. (1996). The caudal-type homeobox protein Cdx-2 binds to the colon promoter of the carbonic anhydrase 1 gene.. Eur J Biochem.

[PDF_00644] Duluc I., Lorentz O., Fritsch C., Leberquier C., Kedinger M., Freund J. N. (1997). Changing intestinal connective tissue interactions alters homeobox gene expression in epithelial cells.. J Cell Sci.

[PDF_00647] Ee H. C., Erler T., Bhathal P. S., Young G. P., James R. J. (1995). Cdx-2 homeodomain protein expression in human and rat colorectal adenoma and carcinoma.. Am J Pathol.

[PDF_00650] Fishel R., Lescoe M. K., Rao M. R., Copeland N. G., Jenkins N. A., Garber J., Kane M., Kolodner R. (1993). The human mutator gene homolog MSH2 and its association with hereditary nonpolyposis colon cancer.. Cell.

[PDF_00654] German M. S., Wang J., Chadwick R. B., Rutter W. J. (1992). Synergistic activation of the insulin gene by a LIM-homeo domain protein and a basic helix-loop-helix protein: building a functional insulin minienhancer complex.. Genes Dev.

[PDF_00658] German M. S., Wang J., Fernald A. A., Espinosa R., Le Beau M. M., Bell G. I. (1994). Localization of the genes encoding two transcription factors, LMX1 and CDX3, regulating insulin gene expression to human chromosomes 1 and 13.. Genomics.

[PDF_00662] Goelz S. E., Hamilton S. R., Vogelstein B. (1985). Purification of DNA from formaldehyde fixed and paraffin embedded human tissue.. Biochem Biophys Res Commun.

[PDF_00665] Groden J., Thliveris A., Samowitz W., Carlson M., Gelbert L., Albertsen H., Joslyn G., Stevens J., Spirio L., Robertson M. (1991). Identification and characterization of the familial adenomatous polyposis coli gene.. Cell.

[PDF_00669] Harvey C. B., Pratt W. S., Islam I., Whitehouse D. B., Swallow D. M. (1995). DNA polymorphisms in the lactase gene. Linkage disequilibrium across the 70-kb region.. Eur J Hum Genet.

[PDF_00674] Irby R. B., Mao W., Coppola D., Kang J., Loubeau J. M., Trudeau W., Karl R., Fujita D. J., Jove R., Yeatman T. J. (1999). Activating SRC mutation in a subset of advanced human colon cancers.. Nat Genet.

[PDF_00679] James R., Erler T., Kazenwadel J. (1994). Structure of the murine homeobox gene cdx-2. Expression in embryonic and adult intestinal epithelium.. J Biol Chem.

[PDF_00677] James R., Kazenwadel J. (1991). Homeobox gene expression in the intestinal epithelium of adult mice.. J Biol Chem.

[PDF_00682] Konishi M., Kikuchi-Yanoshita R., Tanaka K., Muraoka M., Onda A., Okumura Y., Kishi N., Iwama T., Mori T., Koike M. (1996). Molecular nature of colon tumors in hereditary nonpolyposis colon cancer, familial polyposis, and sporadic colon cancer.. Gastroenterology.

[PDF_00687] Lambert M., Colnot S., Suh E., L'Horset F., Blin C., Calliot M. E., Raymondjean M., Thomasset M., Traber P. G., Perret C. (1996). cis-Acting elements and transcription factors involved in the intestinal specific expression of the rat calbindin-D9K gene: binding of the intestine-specific transcription factor Cdx-2 to the TATA box.. Eur J Biochem.

[PDF_00692] Leach F. S., Nicolaides N. C., Papadopoulos N., Liu B., Jen J., Parsons R., Peltomäki P., Sistonen P., Aaltonen L. A., Nyström-Lahti M. (1993). Mutations of a mutS homolog in hereditary nonpolyposis colorectal cancer.. Cell.

[PDF_00695] Li S. S., Tseng H. M., Yang T. P., Liu C. H., Teng S. J., Huang H. W., Chen L. M., Kao H. W., Chen J. H., Tseng J. N. (1999). Molecular characterization of germline mutations in the BRCA1 and BRCA2 genes from breast cancer families in Taiwan.. Hum Genet.

[PDF_00700] Liu B., Nicolaides N. C., Markowitz S., Willson J. K., Parsons R. E., Jen J., Papadopolous N., Peltomäki P., de la Chapelle A., Hamilton S. R. (1995). Mismatch repair gene defects in sporadic colorectal cancers with microsatellite instability.. Nat Genet.

[PDF_00704] Liu B., Parsons R., Papadopoulos N., Nicolaides N. C., Lynch H. T., Watson P., Jass J. R., Dunlop M., Wyllie A., Peltomäki P. (1996). Analysis of mismatch repair genes in hereditary non-polyposis colorectal cancer patients.. Nat Med.

[PDF_00708] Lorentz O., Duluc I., Arcangelis A. D., Simon-Assmann P., Kedinger M., Freund J. N. (1997). Key role of the Cdx2 homeobox gene in extracellular matrix-mediated intestinal cell differentiation.. J Cell Biol.

[PDF_00711] Lothe R. A., Fossli T., Danielsen H. E., Stenwig A. E., Nesland J. M., Gallie B., Børresen A. L. (1992). Molecular genetic studies of tumor suppressor gene regions on chromosomes 13 and 17 in colorectal tumors.. J Natl Cancer Inst.

[PDF_00715] Mallo G. V., Rechreche H., Frigerio J. M., Rocha D., Zweibaum A., Lacasa M., Jordan B. R., Dusetti N. J., Dagorn J. C., Iovanna J. L. (1997). Molecular cloning, sequencing and expression of the mRNA encoding human Cdx1 and Cdx2 homeobox. Down-regulation of Cdx1 and Cdx2 mRNA expression during colorectal carcinogenesis.. Int J Cancer.

[PDF_00720] Mallo G. V., Soubeyran P., Lissitzky J. C., André F., Farnarier C., Marvaldi J., Dagorn J. C., Iovanna J. L. (1998). Expression of the Cdx1 and Cdx2 homeotic genes leads to reduced malignancy in colon cancer-derived cells.. J Biol Chem.

[PDF_00724] Nishisho I., Nakamura Y., Miyoshi Y., Miki Y., Ando H., Horii A., Koyama K., Utsunomiya J., Baba S., Hedge P. (1991). Mutations of chromosome 5q21 genes in FAP and colorectal cancer patients.. Science.

[PDF_00727] Papadopoulos N., Nicolaides N. C., Wei Y. F., Ruben S. M., Carter K. C., Rosen C. A., Haseltine W. A., Fleischmann R. D., Fraser C. M., Adams M. D. (1994). Mutation of a mutL homolog in hereditary colon cancer.. Science.

[PDF_00730] Sham P. C., Curtis D. (1995). Monte Carlo tests for associations between disease and alleles at highly polymorphic loci.. Ann Hum Genet.

[PDF_00732] Sowden J., Leigh S., Talbot I., Delhanty J., Edwards Y. (1993). Expression from the proximal promoter of the carbonic anhydrase 1 gene as a marker for differentiation in colon epithelia.. Differentiation.

[PDF_00735] Suh E., Chen L., Taylor J., Traber P. G. (1994). A homeodomain protein related to caudal regulates intestine-specific gene transcription.. Mol Cell Biol.

[PDF_00737] Tamai Y., Nakajima R., Ishikawa T., Takaku K., Seldin M. F., Taketo M. M. (1999). Colonic hamartoma development by anomalous duplication in Cdx2 knockout mice.. Cancer Res.

[PDF_00740] Wicking C., Simms L. A., Evans T., Walsh M., Chawengsaksophak K., Beck F., Chenevix-Trench G., Young J., Jass J., Leggett B. (1998). CDX2, a human homologue of Drosophila caudal, is mutated in both alleles in a replication error positive colorectal cancer.. Oncogene.

[PDF_00744] Yagi O. K., Akiyama Y., Yuasa Y. (1999). Genomic structure and alterations of homeobox gene CDX2 in colorectal carcinomas.. Br J Cancer.

